# Bioactivity-Directed Isolation of Anticancer Constituents from Underexplored Folklore: *Rhus punjabensis* Stewart

**DOI:** 10.3390/molecules30224339

**Published:** 2025-11-08

**Authors:** Saira Tabassum, Joham Sarfraz Ali, Rida Fatima Saeed, Madiha Asghar, Myra Akhtar, Abdul Momin Rizwan Ahmad, Muhammad Zia

**Affiliations:** 1 National Center for Bioinformatics, Quaid-i-Azam University, Islamabad 45320, Pakistan; saira_tabassum@ymail.com (S.T.); nmadiha608@gmail.com (M.A.); ziachaudhary@gmail.com (M.Z.); 2 Department of Biological Sciences, National University of Medical Sciences (NUMS), Rawalpindi 46000, Pakistan; rida.saeed@numspak.edu.pk; 3 Department of Biological Sciences, International Islamic University, Islamabad 45500, Pakistan; myraakhtar98@gmail.com; 4 Department of Human Nutrition and Dietetics, NUST School of Health Sciences, National University of Sciences & Technology (NUST), Sector H-12, Islamabad 44000, Pakistan; 5 Department of Health Sciences, University of York, York YO10 5DD, UK

**Keywords:** medicinal plant, antioxidant assays, cytotoxicity assays, NF-κB, bioassay guided isolation, bioactive compounds, in vivo

## Abstract

Background: Medicinal plants continue to offer a promising source of novel bioactive compounds for cancer therapy due to their affordability, biocompatibility, and low toxicity. *Rhus punjabensis* Stewart, an ethnomedicinal species from the family Anacardiaceae, has long been used in the traditional medicine of northern Pakistan to treat inflammatory, hepatic, and infectious diseases. However, its phytochemical composition and anticancer potential remain largely unexplored. Methods: This study employed a bioactivity-guided isolation strategy to identify and characterize anticancer constituents from *R. punjabensis* leaves. The plant material was sequentially fractionated using solvents of increasing polarity, followed by purification via column chromatography. Each fraction and purified compound was evaluated using antioxidant (DPPH, total antioxidant capacity, and total reducing power) and cytotoxic assays, including brine shrimp lethality, Sulfo-rhodamine B (SRB) against five human cancer cell lines, protein kinase inhibition, and NF-κB chemo-preventive assays. Results: Comparative analysis of spectral data (UV, 1D/2D NMR, and ESI-MS) led to the identification of three triterpenoid compounds—Lupeol, Cycloartenol, and β-sitosterol—reported for the first time from *R. punjabensis*. Among them, Lupeol displayed the most potent cytotoxicity against DU-145 prostate (IC_50_ = 11.2 ± 1.2 μg/mL) and HL-60 leukemia (IC_50_ = 15.2 ± 1.1 μg/mL) cell lines and showed significant NF-κB inhibitory activity (IC_50_ = 19.4 ± 1.1 μg/mL), indicating its chemo-preventive potential. Cycloartenoland β-sitosterol exhibited moderate antioxidant and antimicrobial activities. Conclusion: The findings validate the ethnopharmacological use of *R. punjabensis* and confirm it as a new source of triterpenoids with notable anticancer activity. This study provides the first comprehensive account of its bioactive metabolites, reinforcing the significance of bioactivity-directed isolation as a powerful approach for discovering natural anticancer agents. Further in vivo and mechanistic evaluations are warranted to establish their therapeutic efficacy and safety profiles.

## 1. Introduction

Cancer remains one of the foremost public health challenges worldwide, causing nearly 10 million deaths annually according to recent World Health Organization [1] estimates. The global burden of cancer continues to rise due to population aging, environmental exposure, and lifestyle-associated risk factors. Conventional chemotherapeutic agents, while effective, are often associated with severe toxicity, resistance, and high cost, prompting a renewed search for safe, cost-effective, and naturally derived alternatives. In this context, medicinal plants have garnered significant scientific attention as a reservoir of structurally diverse compounds that have demonstrated pharmacological activities, including anticancer, antioxidant, and anti-inflammatory effects [2]. Approximately 47% of all cytotoxic drugs approved since the 1940s have either been derived directly from natural products or developed as synthetic analogs of plant-based molecules, underscoring the central role of phytochemistry in modern drug discovery.

The genus *Rhus* L. (family Anacardiaceae) comprises more than 250 species of shrubs and trees distributed across temperate and tropical regions of the world. Several *Rhus* species have been recognized for their ethnomedicinal relevance and have served as traditional remedies for ailments such as fever, liver disorders, inflammation, and infections. Members of the genus—including *Rhus coriaria*, *Rhus verniciflua*, and *Rhus chinensis*—have been extensively studied and reported to contain diverse bioactive constituents, such as flavonoids, polyphenols, and triterpenoids, with strong antioxidant and anticancer properties [3,4,5]. Despite this, *Rhus punjabensis* Stewart, native to the mountainous regions of northern Pakistan, remains largely unexplored in terms of its phytochemical profile and biological activities.

Traditionally, *R. punjabensis* has been employed in local medicine to treat rheumatism, diarrhea, liver complications, and various inflammatory conditions [6,7,8]. Decoctions or pastes prepared from its bark and leaves are used as diaphoretic, cathartic, antirheumatic, and antidiabetic agents. However, its bioactive components and their underlying mechanisms of action remain uncharacterized. The scarcity of comprehensive phytochemical investigations and the lack of systematic evaluation of its cytotoxic and chemo-preventive potential highlight a major research gap that warrants scientific inquiry.

Natural products have historically played a crucial role in the development of anticancer drugs, primarily through the discovery of alkaloids, terpenoids, and polyphenolic compounds with mechanisms involving apoptosis induction, inhibition of metastasis, and modulation of key molecular signaling pathways [9]. Among these, triterpenoids, such as Lupeol, cycloartenol, and β-sitosterol, are of special interest due to their established ability to interfere with tumor progression through modulation of the NF-κB, PI3K/Akt, and MAPK pathways [10,11]. While these compounds have been reported from other *Rhus* species, no prior study has isolated or functionally characterized them from *R. punjabensis*.

Given this context, the present study sought to fill the knowledge gap by employing a bioactivity-guided isolation approach to identify the active constituents responsible for the anticancer and antioxidant properties of *R. punjabensis*. This approach integrates sequential fractionation with biological assays, enabling targeted purification of compounds with the highest pharmacological activity. The leaves of *R. punjabensis* were extracted using solvents of increasing polarity, and the resulting fractions were assessed for antioxidant potential through DPPH, total antioxidant capacity (TAC), and total reducing power (TRP) assays. Their cytotoxic potential was further evaluated through brine shrimp lethality, Sulfo-rhodamine B (SRB) assays against multiple human cancer cell lines, protein kinase inhibition, and NF-κB suppression assays to investigate potential chemo-preventive properties.

The study reports, for the first time, the isolation and structural elucidation of three triterpenoids—Lupeol, Cycloartenol, and β-sitosterol—from *R. punjabensis* using chromatographic and spectroscopic techniques (UV, 1D/2D NMR, and ESI-MS). These compounds demonstrated significant antioxidant and anticancer activities, supporting the ethnopharmacological use of the species and highlighting its promise as a source of novel bioactive leads for cancer therapy. By bridging traditional medicinal knowledge and modern pharmacological validation, this work contributes to the ongoing effort to discover and develop natural product-based anticancer agents from indigenous flora.

To the best of our knowledge, this is the first comprehensive bioactivity-guided isolation and spectroscopic characterization (UV, 1D/2D NMR, and ESI-MS) of Lupeol, Cycloartenol, and β-sitosterol from *Rhus punjabensis*, accompanied by SRB cytotoxicity, NF-κB, and protein kinase modulation assays. While Lupeol and β-sitosterol are known from other species, this is the first report from *R. punjabensis* and the first time their anticancer and NF-κB modulatory activities have been demonstrated in this species.

## 2. Materials and Methods

### 2.1. Plant Material

*R. punjabensis* Stewart was gathered in July 2013 from Lakary Mountain, Shamshaki, District Karak, Khyber Pakhtunkhwa, Pakistan. The location where it was gathered was unruly and did not require governmental approval. The plant was identified by Prof. Dr. Mir Ajab Khan, Department of Plant Sciences (Quaid-i-Azam University, Islamabad, Pakistan). A voucher specimen (HMP 491) was deposited at the Quaid-i-Azam University Herbarium of Medicinal Plants.

### 2.2. Extraction

*R. punjabensis* leaves were rinsed with water, shade-dried at room temperature, and crushed into a fine powder. Ethyl acetate was chosen as a solvent for preparative-scale extraction based on biological activity by macerating a 10 kg powder packaged in tight-headed drums, with each containing 2.5 L of solvent. The extract was separated into fractions using n-hexane (n-hex), a mixture of n-hexane with chloroform (n-hex + CHCl_3_), chloroform (CHCl_3_), a combination of ethyl acetate with n-hexane (EtOAc + n-hex), ethyl acetate (EtOAc), and methanol (MeOH). After immersing for three days, the solvent was strained through a cotton plug and then filtered using a Whatman filter; the residue was extracted three times using the same procedure (Figure S1). The extracts were combined and concentrated in a rotary evaporator (Buchi, Flawil, Switzerland) before being dried in a vacuum oven (Yamato, Tokyo, Japan) at 45 °C. The experiment was repeated thrice, while the fractions were preserved at −30 °C for later use [4].

### 2.3. Isolation of Bioactive Compounds

The EtOAc + n-hex fraction (40.2 g) sourced from NP-CC was still in use. The fraction was liquefied in an appropriate solvent (EtOAc), then dried in a fume hood after being adsorbed on silica gel 60 (230–400 mesh Merck, Darmstadt, Germany) in a ratio of 1 g sample to 1 g silica gel. Next, a glass column was filled with 200 g of silica gel (230–400 mesh, Merck Germany), and the top dried sample was added. Additionally, a “2 cm” silica gel protective covering was applied to the sample’s top. Figure S2 describes a gradient shift in mobile phase that was employed, starting with n-hex: EtOAC in a ratio of 1:0–0:1. Fractions were collected in 300 mL portions during each chromatographic step. A total of 34 fractions were collected for further fractionation. NP-LCC was applied to a mixture of 21–24 fractions utilizing a moving-phase n-hex: EtOAc: 1:0–1:1. Each beaker held a total of 15 fractions (100 mL); fractions 1–8 were mixed and run over a tiny MPLC column using active-phase n-hex: EtOAc: 1:0: 5:1. The sum of 11 parts of the whole was collected. To obtain the pure chemical PEEA-C2, fraction 4 was dried [4].

TLC analysis indicated similarity among fractions 35–37, which were subsequently pooled and processed further for the MPLC column using the mobile phase EtOAc:n-hex:1:0–1:0. For 18 fractions (50 mL), the collection was performed; fractions 5–8 were integrated and directed to small column MPLC, implementing the mobile phase n-hex: EtOAc: 1:0–3:1. A total of 25 mL of 15 fractions each were accumulated, and fractions 7–8 were evaporated to dryness, affording a pure compound designated as PEEA-C3.

The separated fragments, especially ethyl acetate EtOAc (90.2 g) isolated via Normal Phase Chromatography, were utilized; after combining and dissolving into an appropriate solvent using a 1:1 mixture of n-hexane and dichloromethane, they were adsorbed on silica gel 60 using (230–400) mesh silica gel (Merck Germany) in the ratio of 1:1 g sample and dried in a fume hood. Then, a glass column was packed with 250 g/L of silica gel (230–400 mesh Merck Germany), and the dried sample was applied to the top [4].

Figure S3 illustrates the usage of a change in gradient of the mobile phase beginning at n-hex: DCM: 1:0–0:1.4. A 500 mL sample of every fraction was gathered. A total of 41 fractions in all were obtained. Based on TLC profiles, fractions 15–22 were merged and then re-chromatographed using a mobile phase of n-hexane and dichloromethane in a 1:0–1:1 ratio. The fractions 15–22 were combined according to TLC results and then subsequently subjected to column chromatography by using a mobile phase of n-hex: DCM in a 1:0–1:1 ratio.

There were 31 fractions in total (100 mL). Column Chromatography was applied to fraction 21 using a mobile phase of n-hexane: dichloromethane (1:0–5:1). A total of 18 fractions (50 mL) were collected. Fractions 17–21 were merged, and the n-hex: DCM: 1:0–5:1 was used to apply CC. Sixteen fractions, each measuring (25 mL), were combined, with fractions 6–7 undergoing a drying process to obtain pure compound RPEA-C5.

The isolated compounds’ structures were elucidated using spectrophotometric methods (TLC, UV, 1D, 2D NMR, and mass spectrometry). The Bruker 400 MHz spectrometer (Billerica, MA, USA) was used to record 1D and 2D NMR spectra. Chemical changes were measured in parts per million (ppm). The coupling constants (J) are expressed in hertz. In the NMR investigations, Deuterated chloroform (CDCl_3_) was utilized as a solvent. ESI-MS mass spectral data were recorded on an Agilent Technologies instrument [4].

### 2.4. Biological Evaluation

#### 2.4.1. Antioxidant Activity

##### DPPH Assay (FRSA)

The ability of extracts and fractions to scavenge the (DPPH) free radical was used to assess their DPPH free radical scavenging activity [12]. Each sample was diluted to final concentrations of 200, 66.66, 22.22, and 7.41 µg/mL before being combined with DPPH solution (9.2 mg/100 mL in methanol). The absorbance at 515 nm was measured after 30 min of incubation at 37 °C. The following formula was used to calculate percent free radical scavenging activity (%FRSA):

%FRSA = (1 − Abs/Abc) × 100

Abs: absorbance of sample, and Abc: absorbance of negative control.

Ascorbic acid was used as a positive control. IC50 of samples with radical scavenging efficiency > 50% was also calculated.

##### Determination of Total Antioxidant Capacity (TAC)

Total antioxidant capacity was determined using a phosphomolybdenum-based colorimetric assay and was stated as µg AAE/mg extract, as per the protocol suggested by [13] with minor modifications. Each tested sample was combined with the reagent (0.6 M Sulphuric acid, 28 mM Sodium phosphate, and 4 mM ammonium molybdate solution in H_2_O). Ascorbic acid was used as a positive control, and DMSO as a negative control. All tubes were placed at 95 °C and incubated in a water bath for 90 min. The samples were loaded into 96-well plates, and absorbance was recorded at 630 nm with the aid of a microplate reader (Elx 800, BioTek Instrument, Winooski, VT, USA). An ascorbic acid calibration was prepared, and then the corresponding equation (y = 0.0211x + 0.0920, R^2^ = 0.9911) was determined, achieving an R^2^ value of 0.9911.

##### Total Reducing Power Assay (TRP)

Potassium ferricyanide colorimetric experiment was performed to assess the reducing potential of the tested samples using the protocol defined by [14]. A mixture of extracts and phosphate buffer (0.2 mol/L, pH 6.6) and potassium ferricyanide (1% *w*/*v* in H_2_O) was prepared. It was later incubated at 50 °C for 20 min before being treated with trichloroacetic acid (10% *w*/*v* in H_2_O) and centrifuged at 3000 rpm at room temperature for 10 min. The upper layer of the solution was combined with FeCl_3_ (0.1% *w*/*v* in H_2_O). An aliquot of this mixture was transferred to a 96-well plate, and the absorbance was taken at 630 nm. Varying concentrations of ascorbic acid were used to generate a calibration curve (y = 0.037x + 0.7482, R^2^ = 0.9961), and results were represented as µg AAE/mg extract.

### 2.5. Microbial Inhibition Assay

Different bacterial and fungal strains were used to perform antimicrobial assays as per the protocols suggested by [12]. Bacterial strains used in antibacterial assays included Gram-positive strains: *Staphylococcus aureus* (ATCC# 6538) and *Micrococcus luteus* (ATCC# 10240); and Gram-negative strains included *Pseudomonas aeruginosa* (ATCC# 9721), *Bordetella bronchiseptica* (ATCC# 4617), and *Salmonella typhimurium* (ATCC# 14028). All bacterial strains were cultured and maintained on nutrient agar slants at 37 °C. Stock cultures were maintained at 4 °C. Fungal strains used in antifungal assay included *Aspergillus fumigatus* (FCBP# 66), *Fusarium solani* (FCBP# 0291), *Mucor* species (FCBP# 0300), *Aspergillus niger* (FCBP# 0198), and *Aspergillus flavus* (FCBP# 0064), collected from ATCC (American Type Culture Collection). All fungal strains were cultured and maintained on sabouraud dextrose agar at 28 to 30 °C.

### 2.6. Cytotoxicity Assessment

#### 2.6.1. Brine Shrimp Lethality Assay and Antileishmanial Assay

A 24 h lethality test against brine shrimp (*Artemia salina*) larvae was performed on a microplate, as previously described by [15]. Salina eggs (Ocean Star, Galveston, TX, USA) were incubated for 24–48 h in simulated seawater in a specially built two-compartment container under light and warmth (30–32 °C). The mature phototropic nauplii were then harvested and transferred to each well using a Pasteur pipette. Serial concentrations of Doxorubicin and 1% DMSO in seawater were used as positive and negative controls, respectively. After incubation (24 h), living shrimps were counted to calculate the proportion of deaths. Later, the median lethal concentration (LC_50_) of the tested samples was calculated. Stock cultures were maintained at 4 °C. *Leishmania tropica* kwh 23 promastigotes were used for antileishmanial assay. *Leishmania tropica* was cultured in M199 at 25 °C.

#### 2.6.2. Protein Kinase Inhibition Assay

The experiment was carried out with the *Streptomyces 85E* strain using the previously reported protocol by [16] with minor modifications. Streptomyces was revived in sterile tryptone soya broth for 48 h, before being inoculated on Petri plates with minimum ISP4 medium. Each test sample was impregnated on a sterile disk and placed on seeded plates. The plates were incubated at 28 °C for 72 h. The bald zone of inhibition was quantified using a Vernier caliper. Minimum inhibitory concentrations (MICs) at lower values ranging from 50 to 3.12 µg/disk were determined by screening extracts producing an inhibition zone ≥ 10 mm in diameter. Surfactin was used as a positive and DMSO as a negative control, respectively.

#### 2.6.3. In Vitro Cytotoxic Activity

##### Sulfo-Rhodamine B Colorimetric Assay

The assay was performed according to the protocol defined by [17], with a few modifications. The cell lines used for this purpose were MDA-MB-231 (estrogen receptor negative breast cancer cells: ATCC^®^ HTB-26™), PC-3 (prostate cancer: ATCC^®^ CRL-1435™), DU-145 (prostate cancer: ATCC^®^ HTB-81™), HL-60 (Human promyelocytic leukemia cells: ATCC^®^ CCL-240™), and HT-29 (Human Colon Carcinoma Cell Line: ATCC^®^ HTB-38™).

The cells were grown in RPMI-1640/DMEM media supplemented with 5% (*v*/*v*) heat-inactivated fetal bovine serum, amphotericin B, gentamicin, and penicillin at 37 °C in 5% CO_2_. Cells were grown to the necessary confluence level, and appropriate test cell concentrations were prepared with fresh medium to incubate cell solutions in the absence or presence of the test sample, as well as Taxol as a positive control, for 3–4 days in a CO_2_ environment at 37 °C. In a CO_2_ incubator (Panasonic, Kadoma, Japan, MCO-18AC-PE), cells in at least 16 wells were incubated for 30 min at 37 °C in a zero-day control test. Trichloroacetic acid (TCA) was used to fix the cells. After a 30 min incubation at 4 °C, the samples underwent three washes with water. Later, the staining procedure was applied to the living unit for 30 min with sulfo-rhodamine B (SRB), and unbound dye was washed and air-dried. The bound dye was dissolved in Tris base for 5 min on a gyratory shaker (LAB-LINE INSTRUMENT, INC). Absorbance values were determined using a 96-well plate reader (Biotech, Minneapolis, MN, USA, Elx 800).

#### 2.6.4. Chemo-Preventive Assessment

##### NF-κB Assay

The ability of test compounds to interfere with the specific binding between the Biotinylated consensus sequence for the respective factor and the active form of the NF-κB transcription factor was assessed using a commercially available kit (EZ-Detect; TM Transcription Factor Assay System; Pierce Biotechnology-Rockford-IL-USA).

##### NF-κB Evaluation

A cell-based NF-κB procedure was carried out following [18]. HeLa cells were pretreated for 2 h with Rrocaglamide before being stimulated for 40 min with interleukin-1 alpha (IL-1) in black 96-well transparent-bottomed plates. Cells were fixed in 10% formaldehyde (pH 7.2) for 30 min at 25 °C before being rinsed with wash buffer (WB). After that, the cells were treated for 1 h with NF-κB primary and secondary antibodies (Cellomics, Inc., Pittsburgh, PA, USA). Cells were then washed twice with WB after 15 min of incubation with detergent buffer. After adding the NF-κB staining reagent, the plates were covered with aluminum foil and incubated for 1 h at 25 °C in the dark. To measure the suppression of NF-κB translocation from the cytoplasm to the nucleus by active substances, samples were examined under an inverted phase-contrast Axiovert CFL fluorescent microscope equipped with a color camera ProhRess TM C10 Plus (Zeiss, Jena, Germany).

## 3. Results

### 3.1. Biological Evaluation of Fractions Isolated via Solid Phase Extraction

Preliminary evaluation demonstrated significant potential and prompted the selection of *R. punjabensis* leaf extract as the leading candidate for further biological assessment. Based on the previously mentioned assay, EtOAc was employed for bulk extraction.

The choice was based on initial screening results mentioned earlier, which depict promising outcomes, making it an ideal candidate for separation. EtOAc extract of the leaf part underwent solid phase extraction (SPE), yielding six fractions through a chain of solvents with diverse polarities. This assay helps in extracting pharmacologically active substances from selected plants.

### 3.2. Biological Evaluation

#### Percent Free Radical Scavenging Activity, Total Reducing Power Assay, and the Antioxidant Capacity

MeOH exhibited a notable free radical scavenging activity with an IC_50_ value of 11.2 ± 1.77 µg/mL, while an IC_50_ value of 15.5 ± 1.11 µg/mL was recorded for the combination of EtOAc and n-hexane fraction. The observed order of general decline in scavenging capacity was as follows: EtOAc + n-hex > EtOAc > n-hex + CHCl_3_ > CHCl_3_ > n-hex.

**Figure 1:**
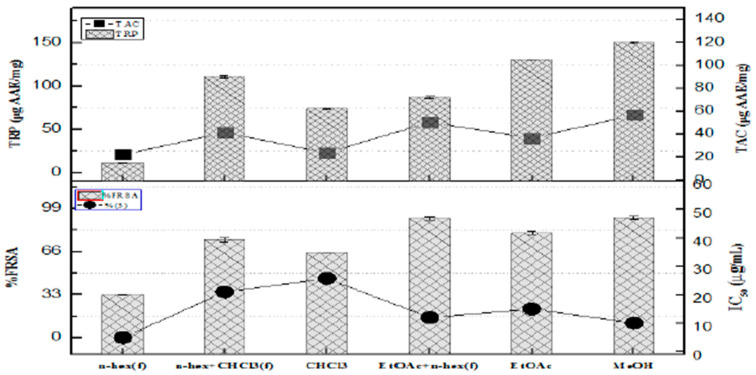
The fraction’s ability to scavenge free radicals, its total reducing power, and overall antioxidant activity.

The MeOH fractions demonstrated the strongest reducing power, with values measuring 150.3 ± 1.27 units. This was followed in order by EtOAc at 130.2 ± 1.11AAE/mg, n-hex + CHC_l3_ combination at 110.8 ± 1.55 µg AAE/mg, and the EtOAc showed a value of (86.5 ± 1.22 µg AAE/mg) while chloroform yielded (74.1 ± 1.11 µg AAE/mg). In contrast, the n-hex fraction depicted the weakest reducing power at 11.2 ± 0.7 µg AAE/mg. The fragments with the highest total antioxidant capacity (TAC) included EtOAc + n-hex (58.2 ± 1.11 µg AAE/mg), n-hex + CHCl_3_ (46.2 ± 1.22 µg AAE/mg), EtOAc (40 ± 1.09 µg AAE/mg), followed by CHCl_3_ (23.2 ± 1.24 µg AAE/mg), and, finally, n-hex (25.5 ± 1.25 µg AAE/mg), as illustrated in Figure 1

### 3.3. Microbial Inhibition Analysis

#### 3.3.1. Antimicrobial and Antifungicidal Properties

This has significantly accelerated the search for novel antimicrobials from alternative sources. Table 1 displays the bactericidal activity of fractions. Except for n-hex and CHCl_3_, all fractions demonstrated significant efficacy against the tested bacterial strains.

Out of all the fractions examined, the EtOAc fraction was shown to be the most active. For *S. typhimurium*, *M. luteus*, *P. aeroginosa*, *S. aureus*, and *B. bronchiseptica*, it demonstrated bactericidal activity with ZOI 24 ± 1.5 mm, 20 ± 1.5 mm, 17.5 ± 1.5 mm, 14.6 ± 1.4 mm, and 14.5 ± 1.5 mm, respectively. ZOI values of 15.6 ± 0.5 mm and 12 ± 0.6 mm, respectively, demonstrated the effectiveness of the EtOAc + n-hex fraction against *S. typhimurium* and *P. aeroginosa*.

The remaining fractions exhibited moderate activity against the microorganisms that were tested. However, the fractions in Table 2 did not show any discernible antifungal properties.

#### 3.3.2. Brine Shrimp Lethality, Antileishmanial Activity, and Assay for Protein Kinase Inhibition Using Fractions Derived from *R. punjabensis*

Several fractions of *R. punjabensis* were assessed for their antileishmanial effects against the *L. tropica* kWh 23 strain. Significant antileishmanial effects were observed for the n-hex fraction with an IC_50_ of 12.6 ± 0.77 µg/mL, then the mixture of n-hexane and chloroform, CHCl_3_, and after that n-hexane, ethyl acetate with n-hex, and, finally, MeOH fractions, which had IC_50_ values of 19.5 ± 0.75, 23.6 ± 1.5, 25.3 ± 1.3, 33.5 ± 1.2, and 40.6 ± 1.4 µg/mL, respectively. Of all the fractions examined, the ethyl acetate with n-hex fractions depicted a notable inhibition zone, measuring 13 ± 1.5 mm, while the n-hex + CHCl_3_ fraction had a zone of inhibition measuring 11 ± 1.7 mm. For reference, Surfactin was employed as a positive control to benchmark the results. Dimethyl sulfoxide, serving as a negative control, demonstrated no cytotoxic effects, validated owing to the absence of any growth inhibition zone. The inhibition zone measurements for the tested samples are presented in Table 3.

#### 3.3.3. In Vitro Cytotoxicity Across Selected Cell Lines

##### Sulfo-Rhodamine B(SRB) Assay

Based on the cytotoxic potential demonstrated by the brine shrimp lethality assay, the plant fractions were evaluated for an in vitro cytotoxic activity across five different cell lines, as shown in Table 4. EtOAc fractions demonstrated significant activity against DU-145 (prostate cancer) and HL-60 (leukemia) cell lines, providing IC_50_ values of 11.1 ± 1.2 μg/mL and 7.03 ± 1.4 μg/mL, correspondingly. Additionally, it exhibited activity against the MDA-MB 231 and PC-3 cell lines with IC_50_ values of 23 ± 1.4 and 35.2 ± 1.1 μg/mL, respectively. Other fractions displayed moderate levels of activity.

##### NF-κB and MTP-Based Chemo-Prevention Assay

The NF-κB signaling pathway may have potential in cancer prevention. The various fractions were assessed by using an inhibition assay (Table 5). The notable findings were represented by a mixture of ethyl acetate and n-hexane, methanol, and n-hexane fractions, which showed the IC_50_ values of 29.4 ± 1.15 μg/mL, 30.5 ± 1.98 μg/mL, and 31.5 ± 1.98 μg/mL, respectively. TNF-α-stimulated nuclear extract was used as a negative control, and Rocaglamide was used as a positive control in this assay.

*R. punjabensis* underwent preliminary assessment via MTP assay, as demonstrated in Table 5. Of them all, the MeOH fraction with (IC_50_ value of 21.6 ± 1.98 μg/mL) had pronounced effects targeting mitochondrial membrane, followed in potency by an n-hex + CHCl_3_ and n-hex alone with an IC_50_ of 30.6 ± 1.98 μg/mL and 31.5 ± 1.98 μg/mL, respectively, while the ethyl acetate with n-hexane fraction exhibited a fairly active IC_50_ of 43.5 ± 1.98 μg/mL. In comparison, Staurosporine had an IC_50_ value of (1.03 ± 1.22 μg/mL).

### 3.4. Characterization of Compounds

The isolated compounds were structurally analyzed using techniques such as mass spectrometry (MS), nuclear magnetic resonance (NMR), and infrared (IR) spectroscopy.

#### 3.4.1. Clarifying the Structure of Penta Cyclic Triterpenoid (PEEA-C2)

Lupeol was obtained as a (9 mg) white, non-crystalline powder. Mass spectrometric analysis was performed, which revealed a molecular ion peak at *m*/*z* 449.5 (M + Na)+. The results from mass spectrometry are shown in Figure S4.

The ^13^C-NMR spectra display chemical shift in (δ 14.8 (Me-27), 15.6 (Me-24), 16.2 (Me-26), 16.3 (Me-25), 18.2 (Me-28), 19.5 (Me-30), and 29.9 (Me-23)). Moreover, nuclear magnetic resonance spectra for PEEA-C2 revealed an oxymethine signal (δH 3.17 (1H, dd, J = 11.0, 5.3 Hz, H-3); δC 79.2) with corresponding isopropanol group signals (δH 4.55 (1H, s, H-29b), 4.67 (1H, s, H-29a), and δC 109.5 (C-29), 151.2 (C-20) in Figure S5.

Figure S6 demonstrated the analysis of ^1^H-NMR of PEEA-C2, which showed the presence of seven methyl groups at δ 0.76 (3H, s, Me-24), 0.81 (3H, s, Me-28), 0.84 (1H, s, ME-25), 0.97 (3H, s, Me-27), 0.98 (3H, s, Me-23), 1.01 (3H, s, Me-26), and 1.70 (3H, s, Me-30). These findings were supported by 13C-NMR spectrum, which displayed peaks at (δ 14.8 (Me-27), 15.6 (Me-24), 16.2 (Me-26), 16.3 (Me-25), 18.2 (Me-28), 19.5 (Me-30), and 29.9 (Me-23).

**Figure 2:**
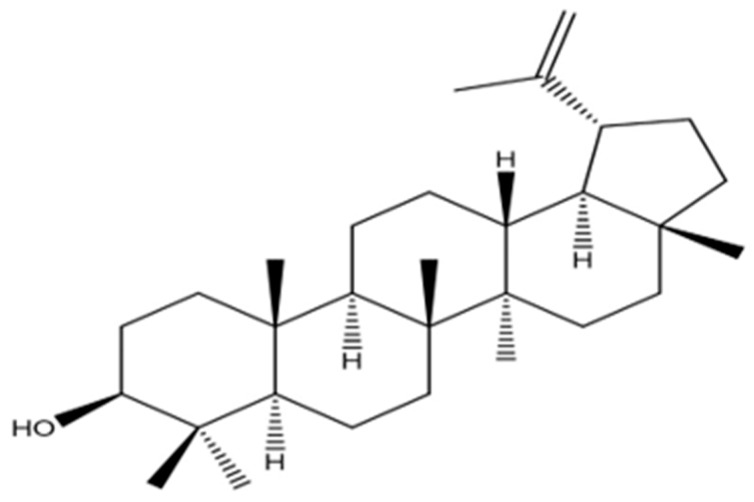
Proposed structure of PEEA-C2 compound.

An oxymethine signal was observed in the NMR spectra of PEEA-C2 as (δH 3.17 (1H, dd, J = 11.0, 5.3 Hz, H-3); δC 79.2). Furthermore, signals for the isopropanol group were observed at (δH 4.55 (1H, s, H-29b), 4.67 (1H, s, H-29a) and δC 109.5 (C-29), 151.2 (C-20)). Analysis of the 13C-NMR DEPT assignments combined with the spectrum established the identification of seven methyl, eleven methylene, six methane, and six quaternary carbons in Figure S7. Ultimately, NMR and physical analyses were consistent with published data for lupeol [19]. The hypothesized molecular structure assigned to PEEA-C2 is demonstrated in Figure 2.

#### 3.4.2. Detailed Structure Determination of PEEA-C3 (Cycloartenol)

The fraction was identified as Petroleum Ether: Ethyl acetate fraction (PEEA-C3) was obtained as 12 mg of needle-like crystals. The molecular mass was assessed using MS, which revealed a molecular ion peak displayed at *m*/*z* 426.72 (M + Na)+ and confirmed a molecular formula of C_30_H_51_O in Figure S8.

Figure S9 illustrates the ^1^H NMR spectrum chemical shifts of δ 1.60 and 1.68. C2: ^1^H-NMR (400 MHz, CDCl_3_): showed the following shift δ 4.96 (m), 4.86 (m), 4.03 (t, J = 8.0 Hz), 3.30 (m), 2.05 (m), 1.90 (m), 1.85 (m), 1.82 (m), 1.77 (m), 1.72 (m), 1.70 (s), 1.65 (m), 1.62 (m), 1.57 (m), 1.50–1.30 (m), 1.01 (s), 0.99 (s), 0.91 (s), 0.80 (s), 0.91 (s), 0.57 (d, J = 4.0 Hz), and 0.35 (d, J = 4.0 Hz). The 13C-NMR (100 MHz, CDCl_3_) data show the following chemical shifts: δ 147.7, 111.3, 78.8, 76.3, 52.1, 48.8, 47.9, 47.1, 45.2, 40.9, 35.5, 32.8, 31.9, 31.2, 30.3, 29.8, 26.4, 26.0, 25.4, 21.1, 19.9, 19.3, 18.3, 18.0, 17.6, 17.2, and 14.0.

**Figure 3:**
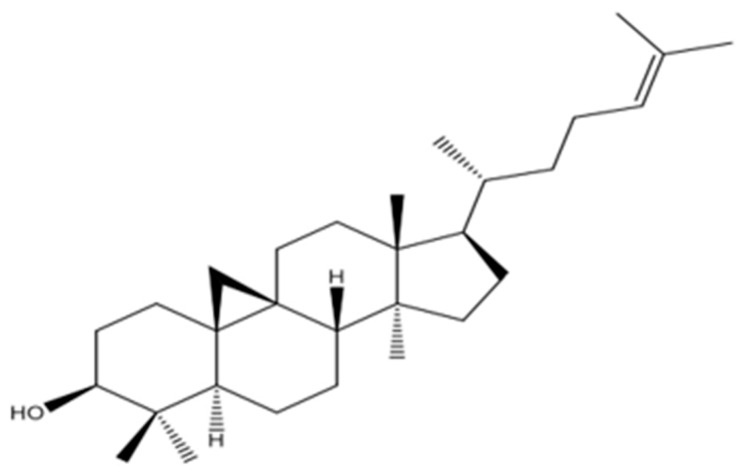
Proposed structure of PEEA-C3 compound.

The chain was validated through 2D-NMR spectra, and structure analysis was conducted based on the correlations found in HMBC and COSY (COSY HSQC, HMBC, DEPT-135), and the COSY spectrum of PEEA-C3 is provided in Figure S10. The compound was named cycloartenol (PEEA-C3), and its structure was clarified and compared with the existing literature [20]. The proposed structure is represented in Figure 3.

#### 3.4.3. Structure Elucidation of RPEA-C5 (β-Sitosterol)

Refined Purified Extract A, Compound 5 (RPEA-C5) was isolated as a white solid weighing 8 mg from the *R. punjabensis* extract. In addition, the molecular formula, viz., C_29_H_50_O, was validated through mass spectroscopy, as illustrated in Figure S11. The ^1^H-NMR spectrum of compound RPEA-C5 showed signals for two tertiary methyls (δ 0.66–0.99), three secondary (δ 0.81,0.830.91), and one primary (δ 0.85) with a corresponding splitting pattern and coupling constant.

**Figure 4:**
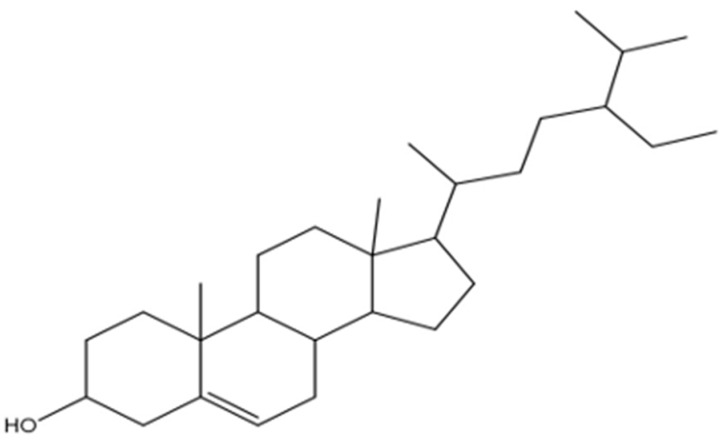
Proposed structure of RPEA-C5 compound.

The findings were further corroborated by ^13^C-NMR analysis, and the peaks were observed for the characteristic methyl group in the range of δ 12.0 to 19.9, corresponding to C-18, C-29, C-27, C-21, C-19, and C-2,6, respectively. Moreover, signals attributed to an oxymethine group were observed in the NMR spectra of compound RPEA-C5 as (δH 3.52 (^1^H, m, H-3); δC 72.0) and olefinic groups (δH 5.34 (1H, m, H-6); δC 140.9 (C-5), 121.9 (C-6)). 13C-NMR spectroscopy supported by broadband decoupling (BB) and DEPT methodologies confirmed that it contains 29 carbon signals, including six methyl, eleven methylene, nine methane, and three quaternary carbon atoms. Ultimately, the structure of compound RPEA-C5 was determined to be β-sitosterol, based on a comparison of its NMR data with the existing literature [16]. The ^13^C NMR spectrum of RPEA-C5 can be found in S 12, along with the proposed structure, as shown in Figure 4.

### 3.5. Antioxidant Activities

#### Total Antioxidant Activity, Total Reducing Power Potential, and Percent Free Radical Scavenging Activity

**Figure 5:**
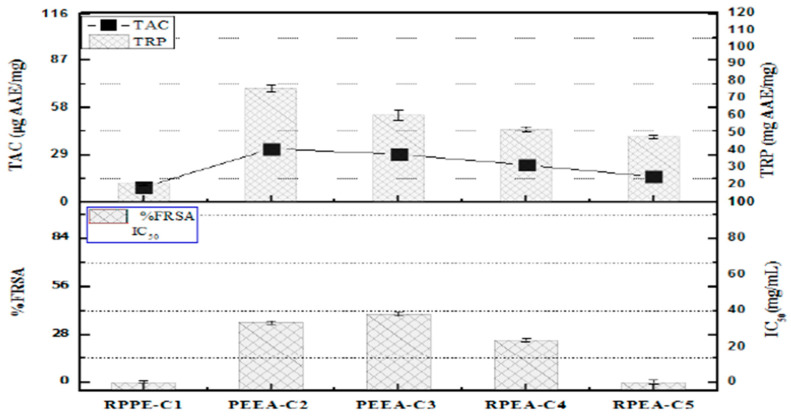
Total antioxidant activity, Free radical scavenging activity, and total reducing power of *R.punjabensis* compounds. Values are presented as mean ± standard deviation from triplicate investigation.

The highest antioxidant potential was depicted by PEEA-C2 (Lupeol) (32.7 ± 1.25 µg AAE/mg), followed by PEEA-C3 (Cycloartenol) (29.4 ± 1.25 µg AAE/mg) and RPEA-C5 (β-sitosterol) (15.7 ± 1.25 µg AAE/mg), respectively. TRP of pure compounds showed the maximum reducing power exhibited by PEEA-C2 (Lupeol) (69.9 ± 2.25 µg AAE/mg compound), followed by PEEA-C3 (cycloartenol) (53.5 ± 1.65 µg AAE/mg compound) and RPEA-C5 (β-sitosterol) (40.1 ± 1.25 µg AAE/mg compound)%FRSA of test samples. The evaluation, based on the discoloration of the DPPH solution, indicated that the compounds showed minimal free radical scavenging activity, with scavenging percentages of 40%, 35%, and 25% for PEEA-C3 (Cycloartenol), PEEA-C2 (Lupeol), and RPEA-C4, respectively. In contrast, RPEA-C5 (β-sitosterol) displayed no activity. All results are presented in Figure 5.

### 3.6. Antimicrobial Assessment

#### 3.6.1. Antibacterial and Antifungal Activity

The antifungal pharmacological effect displayed by the substance is presented in Table 6 Each of the five chemical entities showed intermediate outcomes against the examined fungal isolates. PEEA-C2 (Lupeol) displayed a susceptibility zone of 7.5 ± 0.7 mm against *A. niger*, and compound PEEA-C3 (Cycloartenol) displayed a 7.5 ± 0.8 mm zone against *F. solani*. Compound RPEA-C5 (β-sitosterol) was found to be moderately active against *A. niger* and *A. flavus* with ZOI 7.5 ± 0.8 mm and 7.5 ± 0.7 mm. The data obtained were compared to the Terbinafine positive control shown in Table 7.

#### 3.6.2. Antileishmanial Activity, Protein Kinase Inhibition Assay, and Brine Shrimp Lethality

Protein Kinase Inhibition screening, with the results of bacterial suppression measured for pure compounds, showed that of the three pure compounds, PEEA-C2 (Lupeol) showed a significant inhibition zone of 13.5 ± 1.7 mm area of bacterial inhibition, followed by the PEEA-C3 (Cycloartenol) 12.5 ± 1.6 mm zone.

The compound, i.e., RPEA-C5 (β-sitosterol), had the lowest activity with the zone of inhibition being 6.5 ± 0.8 mm. These values were contrasted with the positive control Surfactin. The cytotoxicity potential of the compounds was subjected to analysis against brine shrimp, as depicted in Table 8.

### 3.7. Cytotoxic Activities Against Different Cancer Cell Lines (Sulforhodamine B Assay)

All compounds following purification were subsequently evaluated for non-clinic cytotoxic screening across five distinct cell lines, as outlined in Table 9. Of the three constituents tested, Lupeol demonstrated notable activity against the human prostate carcinoma (DU-145) and human promyelocytic leukemia (HL-60) cell lines, exhibiting IC_50_ values of 11.2 ± 1.2 μg/mL and 15.2 ± 1.1 μg/mL, respectively. Furthermore, the PEEA-C2 (Lupeol) compound also showed significant efficacy against MDA-MB-231 and PC-3 cell lines, with IC_50_ values of 29.4 ± 1.1 and 31.2 ± 1.4 μg/mL, respectively. PEEA-C3 (Cycloartenol) was found to exert cytotoxic effects on MDA-MB 231, yielding an IC_50_ value of 31.2 ± 1.3 μg/mL. In contrast, compound RPEA-C5 (β-sitosterol) exhibited moderate activity against the DU-145 cell line and HL-60, with IC_50_ values of 29.5 ± 1.7 μg/mL and IC_50_ values of 25.3 ± 1.3 μg/mL against the cell line. All compounds except for PEEA-C2 (Lupeol) demonstrated negligible activity against prostate cancer cell lines (PC-3). These results were compared with Taxol (positive control).

#### Cancer Chemo Preventative Assessments by Using NF-κB and MTP Assays

The Nuclear Factor Kappa Light Chain Enhancer of activated B cells (NF-κB) signaling pathway holds promise as a preventive measure for cancer. Five different compounds were evaluated using an NF-κB inhibition assay, as shown in Table 10. A nuclear extract of HeLa cells treated with each test sample and TNF-α was used for the evaluation of specific binding. TNF-α-stimulated nuclear extract was used as a negative control, and Rocaglamide as a positive control in this assay. The PEEA-C2 (lupeol) compound demonstrated the strongest NF-κB inhibitory activity, yielding an IC50 value of 19.4 ± 1.1 μg/mL. Meanwhile, the PEEA-C3 (Cycloartenol) and RPEA-C5 (β-sitosterol) compounds exhibited activities that were not statistically significant. These findings were comparable to the effects observed with the positive control, Rocaglamide. The MTP assay indicated a swift rise in intracellular calcium levels along with a rapid loss of mitochondrial membrane potential. Multiple secondary metabolites isolated from *R. punjabensis* were quantified using the microtiter plate (MTP) assays shown in Table 10. Within the set of three, PEEA-C2 (Lupeol) demonstrated significant activity, showing an IC50 value of 28.6 ± 1.9 μg/mL against the mitochondrial membrane. In contrast, RPEA-C5 (β-sitosterol) displayed minimal activity with an IC_50_ value of 41.5 ± 1.1 μg/mL. These findings were consistent with those observed for Staurosporine.

## 4. Discussion

These natural products, particularly the secondary metabolites, have been extensively studied and have shown anticarcinogenic activity by interfering with the initiation, development, and progression of cancer via various mechanisms, such as cellular proliferation, apoptosis, differentiation, angiogenesis, and metastasis. This idea is becoming more widely embraced, as it provides a budget-friendly option for cancer care [9].

In the current study, we assessed six fractions of *R. punjabensis* leaves’ ethyl acetate extract using bioassay-guided isolation. Three compounds were isolated using column chromatography, and to the best of my knowledge, this is the first time they have been reported using bioassay-guided isolation from *R. punjabensis*. The antioxidant activities of various fractions and compounds in this research were assessed by utilizing several antioxidant assays, such as free radical scavenging, total reducing power, and total antioxidant capacity assays. The scavenging of DPPH-generated free radicals was used to determine the %FRSA of specimens. The current scavenging results support previous findings in another allied species of Rhus. *R. vericiflua* stroke exhibits notable antioxidant and antitumor characteristics, alongside the ability to counteract Fe+2-induced linoleic acid peroxidation [4,21]. Previous research has shown that polar extracts have a strong reducing power [22]. Plants can provide natural antioxidants, phytochemicals, and secondary metabolites [23]. A positive association has been discovered between reducing power and antioxidant potential, specifically in moderately polar solvents, which is consistent with previous results [24].

Triterpenes are naturally occurring components of human diets. An average of 250 mg/day of triterpenes is ingested in the West and other countries, primarily from vegetable oils, grains, fruits, and vegetables [25]. Consistent with prior reports, Lupeol (PEEA-C2) is a triterpenoid that has been discovered in a variety of plants from phylogenetically diverse families. It has received a lot of research due to its robust and diversified biological activity and anticancer potential [12]. In the current study, PEEA-C2 (Lupeol) has shown substantial antioxidant activity in comparison to prior findings. The effect of DPPH (2, 2-Diphenyl-1-Picryl Hydrazyl) revealed that Lupeol had a higher percentage of antioxidant activity than ascorbic acid at higher concentrations. TRP (total reducing power) results were in support of DPPH. Lupeol is well known for its use as an oxidation-mitigating agent [10]. Because of its propensity to give electrons to hydrogen peroxide, lupeol efficiently scavenged hydrogen. According to the findings of this study, Lupeol has a high free radical scavenging activity in the human body and can be considered a potent and safer compound to be used to cure various ailments, including cancer [26].

The brine shrimp cytotoxicity assay determines the cytotoxic activity of herbal compounds targeting recently emerged larvae of *Artemia saline* [4]. The brine shrimp toxicity test has been frequently used to investigate antibacterial and anticancer [27] properties that combat malaria, fungi, larvae, mollusks, and insects [28]. For the first time, the present screening was used to test the fractions and compounds of *R. punjabensis*. The death rate of brine shrimp reduces as the concentration diminishes, and the level of lethality primarily depends on the concentration itself. The larvicidal property of *R. punjabensis* p-ether fraction could be attributable to the presence of several cytotoxic components, as determined by bioassay-guided isolation. The compound RPEA-C5 (β-sitosterol) demonstrated significant activity. The considerable activity of the fractions is most likely due to the combination of solvents used in the activity, which is evident from its current use in chemo-preventive drugs [26].

Protein kinase inhibitors are a major subset of oncogenic kinase inhibitors. Protein kinases phosphorylate proteins on serine/threonine and tyrosine residues, which are implicated in major regulatory mechanisms in biological processes such as apoptosis, cell proliferation, cell differentiation, and metabolism [29]. The occurrence of genetic changes during early tumor formation can result in unregulated phosphorylation associated with these pathways, ultimately leading to cancer progression. In this line, researchers all around the world are interested in identifying kinase inhibitors, which could result in the formation of new tools for chemo-prevention [30]. Shangbhag et al. [31] have reported that the earlier discovery of the production of *Streptomyces* sp. *(85E)* aerial hyphae requires protein kinase activity, which can be inhibited by several kinase inhibitors. The findings suggest that *R. punjabensis* has the potential to engage allosterically with active or inactive sites of certain kinases and pharmacologically limit cancer growth. The considerable activity of the ethyl acetate fractions. The new findings help evaluate cytotoxicity against more cancer cell lines.

The cytotoxicity of five fractions and three compounds was assessed using a colorimetric assay with Sulfo-rhodamine B (SRB). The most effective fraction against DU-145 and HL-60 was p-ether + EtOAc. Based on bioassay-guided isolation, PEEA-C2 (Lupeol) was found to be very potent against DU-145 and HL-60. Our findings are supported by agreements made by other Rhus species, *R. succedanea* L. Recent studies have shown that various compounds exhibit notable cytotoxic effects against five diverse malignant cell-based systems, which include the liver cell line (Huh7), lower uterine epithelioid carcinoma (HeLa), malignant glandular tumor of the colon (LoVo), gastrointestinal epithelial cancer cell lines (HCT116), and rat C6 glioma cells [5]. These results align with the existing literature that highlights the significant cytotoxic and anticancer potential of *R. chinensis* Mill [32] and *R. verniciflua* Stokes against mouse embryonic primary hepatic cells (MPHC), embryonic normal hepatic cell line (BNL CL.2), and the SV40-mediated transformed cell line (BNL SV A.8) [33].

The NF-κB pathway regulates the expression of several oncogene and tumor suppressor genes (c-myc, p53), genes encoding cell adhesion proteins (VCAM-1, ELAM-1, ICAM-1), and extracellular matrix proteases [34]. NF-κB is activated by a variety of substances, including growth factors, carcinogens, and tumor promoters such as TPA [35]. According to research, NF-κB activity affects cell survival and defines cancer cell sensitivity to cytotoxic drugs as well as ionizing radiation [36].

The NF-κB signaling pathway may be useful in the prevention or treatment of cancer. In the current study, *R. punjabensis* EtOAc fractions, as well as isolated compounds, demonstrated a substantial NF-κB pathway. The current work, which is supported by the previous literature, shows that *R. coriaria* extract inhibits TNBC migration and invasion, suppresses angiogenesis, and lowers tumor growth in vivo via inhibiting STAT3, NF-κB pathways. The *R. coriaria* extract significantly suppressed p65 phosphorylation, as well as its ability to inhibit NF-κB transcriptional activity [37]. Taken together, the data clearly show that *R. coriaria* extract exerts its effects on breast cancer cell proliferation, migration, and invasion in part by inhibiting two key signaling pathways, namely STAT3 and NF-κB, which are known to regulate several processes in breast cancer, including tumor growth and metastasis [38].

According to reports, the lower risk of several malignancies associated with frequent olive oil intake may be linked to its high triterpene concentration [39]. When tested against several cancer cell lines, several triterpenoids have shown potential as antineoplastic drugs and antiproliferative action [40]. Members of the cycloartane, lupane, dammarane, friedelane, oleanane, ursane, limonoid, and cucurbitacin families are included in this group [41]. Many studies have shown that triterpenes directly reduce cytotoxicity, cell cycle progression, and tumor growth, and induce death in tumor cells in vitro and in vivo [42]. Lupeol has been shown to have considerable antimutagenic effects in both in vitro and in vivo systems [43]. Lupeol’s antitumor promotion effects have been linked to its ability to adjust the signaling pathways like the NF-κB pathway and the PI3K/Akt (protein kinase B) pathway, both of which have been linked to tumor genesis [44].

As a result, the significant antiproliferative activity in our current investigation can be attributed to many compounds that are abundantly present in this plant and bio-guided isolation as well, which exhibited the same close connection with the fraction’s activities and compound activities. These are cancer treatment strategies and, in turn, they aid individuals fighting this deadly disease.

For comprehensive chemotaxonomic profiling and de-replication, future work will employ LC-MS/MS coupled with molecular-networking (e.g., GNPS) to map related metabolites across *Rhus* species and facilitate rapid identification of minor constituents.

Planned follow-up studies include in vivo efficacy and toxicity assessments (murine xenograft models for prostate cancer and leukemia) and pathway-specific assays to delineate the mechanisms underlying NF-κB suppression.

## 5. Conclusions

The present study provides a comprehensive bioactivity-guided investigation of *Rhus punjabensis* Stewart, revealing its potential as a new source of bioactive triterpenoids with significant antioxidant, antimicrobial, and anticancer properties. Sequential extraction and chromatographic purification of the chloroform fraction led to the isolation of three major compounds—Lupeol, Cycloartenol, and β-sitosterol—identified for the first time from this plant through combined spectroscopic analyses (UV, IR, NMR, and ESI-MS).

Among the isolated compounds, Lupeol demonstrated the most potent bioactivity, exhibiting marked cytotoxicity against DU-145 prostate and HL-60 leukemia cell lines in both MTT and SRB assays, alongside strong NF-κB inhibition, indicating its chemo-preventive and apoptosis-inducing potential. Cycloartenol and β-sitosterol displayed moderate antioxidant and antimicrobial effects, supporting their contributory role in the overall bio efficacy of *R. punjabensis* extracts. These results collectively validate the traditional medicinal use of the plant in managing inflammation and related pathologies and highlight triterpenoids as the principal bioactive constituents responsible for its therapeutic activity.

The study is novel in being the first to isolate and characterize these triterpenoids from *R. punjabensis* and to correlate them systematically with biological assays targeting oxidative stress, microbial resistance, and cancer cell proliferation. The findings underscore the significance of bioactivity-directed fractionation as an effective approach for natural product discovery and emphasize *R. punjabensis* as a promising candidate for future drug development pipelines focused on triterpenoid scaffolds.

However, while the in vitro data strongly support the anticancer potential of these metabolites, the study’s main limitation lies in the absence of in vivo validation and mechanistic exploration. Further research involving animal models, molecular docking, gene expression profiling, and pharmacokinetic evaluation is warranted to elucidate the precise mechanisms underlying their cytotoxic and NF-κB inhibitory effects.

In conclusion, this investigation not only advances phytochemical knowledge of *Rhus punjabensis* but also contributes valuable insight into the anticancer potential of its triterpenoid constituents. By linking traditional medicinal knowledge with contemporary biochemical validation, the study establishes a scientific foundation for the development of triterpenoid-based chemo-preventive agents derived from indigenous plant sources.

## Figures and Tables

**Table 1:** Antibacterial activities of fractions, in terms of zone of inhibition (ZOI) and their MICs.

Samples	*P. aeruginosa*	MIC	*S. typhimurium*	MIC	*M. luteus*	MIC	*B. bronchiseptica*	MIC	*S. aureus*	MIC
	ZOI (mm)	µg/mL	ZOI (mm)	µg/mL	ZOI (mm)	µg/mL	ZOI (mm)	µg/mL	ZOI (mm)	µg/mL
n-hex (f)	7 ± 0.7	--	7.5 ± 0.5	--	7.5 ± 0.5	--	7 ± 0.7	--	7.7 ± 0.7	--
n-hex + CHCl_3_ (f)	11.0 ± 0.8 *	--	10.5 ± 0.7 *	33.3	12.5 ± 0.5 *	33.3	10.5 ± 0.7	--	7.7 ± 0.7	--
CHCl_3_ (f)	8.5 ± 0.7	--	9 ± 0.5	--	7.5 ± 0.5	--	7.5 ± 0.8	--	7.5 ± 0.8	--
EtOAc + n-hex (f)	12 ± 0.6	33.3	15.6 ± 0.5 **	33.3	11 ± 0.7 *	33.3	10 ± 0.7 *	33.3	7.5 ± 0.7	--
EtOAc (f)	17.5 ± 1.5 **	11.1	24 ± 1.5 ***	1.11	20 ± 1.5 ***	11.3	14.5 ± 1.5 **	11.3	14.6 ± 1.4 **	33.3
MeOH (f)	10.5 ± 0.7 *	--	1 1 ± 0.5 *	33.3	6.5 ± 0.7	--	7.5 ± 0.5	--	6 ± 0.4	--
Roxithromycin	22.5 ± 2.5 ***	0.1	25 ± 2.3 ***	0.1	22.5 ± 2.8 ***	0.1	18 ± 2.8 ***	0.1	20 ± 2.2 ***	0.1
DMSO	--	--	--	--	--	--	--	--	--	--

* ZOI: zone of inhibition (mm) at 100 µg/disk. Values (mean ± SD) are the average of three samples, analyzed individually in triplicate (*n* = 1 × 3). -- = No activity in antibacterial assay or not active (zone ˂ 10 mm) for MIC. Results are highly significant ***, moderately significant **, and significant *.

**Table 2:** Evaluating the antifungal effectiveness of tested fractions on filamentous fungi.

Samples	*A. fumigatus*	*Mucor* Species	*A. niger*	*F. solani*	*A. flavus*
n-hex (f)	6.5 ± 0.5	7.5 ± 0.5	7 ± 0.7	7.5 ± 0.7	7.5 ± 0.7
n-hex + CHCl_3_ (f)	6.5 ± 0.6	6.5 ± 0.7	7.5 ± 0.5	7.5 ± 0.8	7.5 ± 0.7
CHCl_3_ (f)	7.5 ± 0.7	7 ± 0.85	6.5 ± 0.6	7.5 ± 0.5	6 ± 0.5
EtOAc + n-hex (f)	7.5 ± 0.5	--	--	7.5 ± 0.8	--
EtOAc (f)	7.5 ± 0.7	--	7.5 ± 0.5	7.5 ± 0.7	--
MeOH (f)	6 ± 0.7	--	7.5 ± 0.5 *	7.5 ± 0.9 *	7.5 ± 0.8 *
Terbinafine	25.5 ± 2.5 ***	24 ± 2.2 ***	23 ± 2.1 ***	25 ± 2.4 ***	25 ± 2.4 ***
DMSO	--	--	--	--	--

* Zone of inhibition (mm) at 100 µg/ disk. Values (mean ± SD) are the average of three samples analyzed individually in triplicate (*n* = 1 × 3). -- = No activity, highly significant ***, and significant *, respectively.

**Table 3:** Brine shrimp lethality, antileishmanial activity, and protein kinase inhibition of fractions from *R. punjabensis*.

Brine Shrimp Lethality		LC_50_	Inhibition of Leishmanial Promastigotes		Protein Kinase Inhibition	
% Mortality at 200 µg/mL		(μg/mL)	% Inhibition		Diameter (mm) at 100 µg/Disk	
					Clear Zone	Bald Zone
Samples						
n-hex (f)	98 ± 9.0 ***	36.7 ± 1.9	95± 1.1 ***	12.6 ± 0.77	7 ± 0.5	--
n-hex + CHCl_3_ (f)	71 ± 8.0 ***	52.2 ± 1.6	90 ± 1.7 ***	19.5 ± 0.75	--	11 ± 1.7 ***
CHCl_3_ (f)	98 ± 9.0 ***	38.5 ± 1.8	85 ± 1.2 *	23.6 ± 1.5	6 ± 0.6	--
EtOAc + n-hex (f)	86 ± 6.1 *	64.5 ± 1.4	88 ± 1.3 **	25.3 ± 1.3	--	13 ± 1.5 ***
EtOAc (f)	70 ± 8.1	58.7 ± 1.5	75 ± 0.9 *	33.5 ± 1.2	--	--
MeOH (f)	75 ± 6.6	62.7 ± 1.3	65 ± 0.9	40.6 ± 1.4	10 ± 1.7 ***	--
Surfactin	--	--			--	17 ± 1.7 ***
Amphotericin-B			100 ± 1.1 ***	1.36 ± 1.5		
Doxorubicin	100 ± 9.1 ***	5.36 ± 2.0				--
DMSO	--	--			--	--

* Zone of inhibition, including the diameter of the disk (5 mm). Data was analyzed individually in triplicate (n = 1 × 3). --: no activity. Doxorubicin was the positive control for the brine shrimp assay. Surfactin was a positive control for the protein kinase inhibition assay. highly significant ***, moderately significant **, and significant *, respectively.

**Table 4:** SRB cytotoxic potential of fractions from *R. punjabensis* against different cell lines.

	DI-145		HL-60		MDA-MB231		HT-29		PC-3	
Samples	% Inhibition	IC_50_	% Inhibition	IC_50_	% Inhibition	IC_50_	% Inhibition	IC_50_	% Inhibition	IC_50_
	at 20 µg/mL	µg/mL	at 20 µg/mL	µg/mL	at 20 µg/mL	µg/mL	at 20 µg/mL	µg/mL	at 20 µg/mL	µg/mL
n-hex (f)	76 ± 1.6 **	22.2 ± 1.2	70 ± 1.2 **	15.21 ± 1.1	65 ± 1.6	45.2 ± 1.8	45 ± 0.7	--	52 ± 1.1	45.1 ± 0.2
n-hex + CHCl_3_	60 ± 1.5	--	75 ± 1.2 **	20.5 ± 1.5	80 ± 1.5 ***	22.5 ± 1.9	60 ± 1.1	41.2 ± 1.7	44 ± 0.6	--
CHCl_3_ (f)	75 ± 1.4 *	21.2 ± 1.3	40 ± 1.4	--	40 ± 1.2	--	56 ± 0.4	45.2 ± 1.5	50 ± 1.6	--
EtOAc + n-hex (f)	76 ± 1.6 **	19.5 ± 1.7	70 ± 1.9 **	17.03 ± 1.1	65 ± 1.5	45.2 ± 1.9	45 ± 0.1	51.2 ± 1.8	52 ± 1.1	45.2 ± 1.9
EtOAc (f)	85 ± 1.4 ***	11.1 ± 1.2	85 ± 2.2 ***	7.03 ± 1.4	75 ± 1.1 **	23 ± 1.4	40 ± 0.1	--	60 ± 1.6 *	35.2 ± 1.1
MeOH	60 ± 1.1	--	75 ± 1.2 **	20.5 ± 1.5	80 ± 1.8 ***	25.5 ± 1.9	60 ± 1.3 *	41.2 ± 1.7	44 ± 0.6	--
Taxol	92 ± 1.6 ***	3.21 ± 1.4	91 ± 1.1 ***	2.88 ± 1.2	91 ± 1.2 ***	3.44 ± 1.2	95 ± 2.5 ***	2.9 ± 1.2	95 ± 2.5 ***	3.8 ± 1.2

Data shown in triplicate ± SD. --: no activity. Means difference is highly significant ***, slightly significant **, and significant *.

**Table 5:** Cancer chemo-preventive assays (NF-κB and MTP) of fractions.

Samples	NF-κB		MTP	
	% Inhibition	IC_50_	% Inhibition	IC_50_
	at 20 μg/mL	(μg/mL)	at 20 μg/mL	(μg/mL)
n-hex (f)	75 ± 1.24 *	31.5 ± 1.98	75 ± 1.12 ***	31.5 ± 1.98
n-hex + CHCl_3_ (f)	68 ± 1.97 *	--	75 ± 1.24 **	30.6 ± 1.98
CHCl_3_ (f)	50 ± 0.17 *	--	65 ± 1.56 *	--
EtOAc + n-hex (f)	86 ± 1.97 **	29.4 ± 1.15	65 ± 1.24 *	43.5 ± 1.98
EtOAc (f)	70 ± 1.97 **	--	60 ± 1.15 *	--
MeOH (f)	75 ± 1.24 **	30.5 ± 1.98	80 ± 1.24 **	21.6 ± 1.98
Rocaglamide	100 ± 3.12 ***	0.03 ± 1.13	--	--
Staurosporine	--		95 ± 1.15 ***	1.03 ± 1.22

* Data shown in triplicate ± SD. --: no activity. Results are highly significant ***, moderately significant **, and significant *, respectively.

**Table 6:** Antibacterial potentials of isolated compounds are assessed through the zone of inhibition (ZOI) and MIC determination.

Samples	*P. aeruginosa* ZOI (mm)	MIC	*S. typhimurium* ZOI (mm)	MIC	*M. luteus* ZOI (mm)	MIC	*B. bronchiseptica* ZOI (mm)	MIC	*S. aureus* ZOI (mm)	MIC
PEEA-C2	16 ± 1.5 ***	11.1	10 ± 0.8	100	14.5 ± 1.7 ***	11.1	13.5 ± 1.5 **	11.3	14 ± 1.7 ***	33.3
PEEA-C3	11.0 ± 1.5	--	12.5 ± 1.7 **	--	12.5 ± 1.5 **	33.3	10.5 ± 1.2	--	7.7 ± 0.9	100
RPEA-C5	10.5 ± 0.7	--	11 ± 1.8 *	100	6.5 ± 0.6	100	7.5 ± 0.8	--	6 ± 0.6	--
Roxithromycin	18.5 ± 2.9 ***	1.1	16 ± 2.3 ***	1.1	26.5 ± 2.4 ***	0.1	22.5 ± 2.5 ***	0.1	21 ± 2.3 ***	0.1
DMSO	--	--	--	--	--	--	--	--	--	--

* Zone of inhibition (mm) at 100 µg/disk. Values (mean ± SD) are the average of three samples. -- = No activity in antibacterial assay or not active (zone ˂ 10 mm) for MIC determination. Results are highly significant ***, moderately significant **, and significant *.

**Table 7:** Antifungal activity of compounds was evaluated and tested against fungal species.

Samples	*A. fumigatus* ZOI (mm)	*Mucor* Species ZOI (mm)	*A. niger* ZOI (mm)	*F. solani* ZOI (mm)	*A. flavus* ZOI (mm)
PEEA-C2	--	--	7.5 ± 0.7 *	--	--
PEEA-C3	7.5 ± 0.6 *	--	--	7.5 ± 0.8 *	--
RPEA-C5	--	--	7.5 ± 0.8 *	--	7.5 ± 0.7 *
Terbinafine	25.5 ± 2.5 ***	24 ± 2.2 ***	23 ± 2.1 ***	25 ± 2.4 ***	25 ± 2.4 ***
DMSO	--	--	--	--	--

* ‘ZOI’ Zone of inhibition (mm) at 100 µg/disk. Values (mean ± SD) are the average of three samples. -- = No activity in antifungal assay. Highly significant ***, and significant *.

**Table 8:** Brine shrimp lethality, antileishmanial, and protein kinase inhibitory activities of isolated compounds.

Samples	Brine Shrimp Lethality	LC_50_	Inhibition of *leishmanial* Promastigotes	IC_50_	Protein Kinase Inhibition	
	% Mortality at 200 µg/mL	(μg/mL)	% Inhibition	(μg/mL)	* Diameter (mm) at 100 µg/Disk	
					Clear Zone	Bald Zone
PEEA-C2	82 ± 8.7 **	23.1 ± 1.2	82.43 ± 6.8 **	16.2 ± 1.7	13.5 ± 1.7 ***	
PEEA-C3	76 ± 7.2 *	33.9 ± 1.2	70 ± 6.7 *	36.9 ± 1.2	--	12.5 ± 1.6 ***
RPEA-C5	60 ± 7.2	44.9 ± 1.5	65 ± 6.1	40.1± 1.2	--	6.5 ± 0.8
Doxorubicin	100 ± 9.1 ***	5.32 ± 2.0			--	
Amphotericin-B			100 ± 8.1 ***	1.36 ± 0.1		
DMSO	--	--	--	--	--	--
Surfactin						17 ± 1.02 ***

* Zone of inhibition, including the diameter of the disk (5 mm). Data was analyzed individually in triplicate (*n* = 1 × 3). Doxorubicin was used as a positive control for the brine shrimp assay, and surfactin as a positive control for the protein kinase inhibition assay. Results are highly significant ***, moderately significant **, and significant *.

**Table 9:** Cytotoxic effects of isolated compounds from *R. punjabensis* using the *Sulforhodamine B assay*.

Samples	DU-145		HL-60		MDA-MB231		HT-29		PC-3	
	% Inhibition	IC_50_	% Inhibition	IC_50_	% Inhibition	IC_50_	% Inhibition	ICI_50_	% Inhibition	IC_50_
	at 20 μg/mL	(μg/mL)	at 20 μg/mL	(μg/mL)	at 20 μg/mL	(μg/mL)	at 20 μg/mL	(μg/mL)	at 20 μg/mL	(μg/mL)
PEEA-C2	90 ± 1.7 ***	11.2 ± 1.2	89 ± 1.7 **	15.2 ± 1.1	70 ± 1.7 *	29.4 ± 1.1	65± 2.5 *	--	75 ± 1.7 *	31.2 ± 1.4
PEEA-C3	80 ± 2.6 **	21.7 ± 1.4	85 ± 1.5 **	21.3 ± 1.6	75 ± 1.9 *	31.2 ± 1.3	65 ± 1.7 *	41.2 ± 1.4	40 ± 1.5	
RPEA-C5	76 ± 1.6 *	29.5± 1.7	70 ± 1.9 *	25.3 ± 1.3	65 ± 1.6	--	45 ± 0.1	--	52 ± 1.1	
Taxol	98 ± 2.8 ***	1.54 ± 1.1	100 ± 3.6 ***	1.7 ± 1.6	95 ± 2.5 **	1.6 ± 1.2	95 ± 2.5 ***	2.9 ± 1.2	95 ± 2.5 **	3.8 ± 1.2

* Data is shown in triplicate as means ± standard error. IC_50_ = concentration at 50% inhibition. -- = not active. Results are highly significant ***, moderately significant **, and significant *.

**Table 10:** Evaluation of isolated compounds for cancer chemo-prevention (NF-κB and MTP).

Samples	NF-κB		MTP	
	% Inhibition	IC_50_	% Inhibition	IC_50_
	at 20 μg/mL	(μg/mL)	at 20 μg/mL	(μg/mL)
PEEA-C2	80 ± 1.7 **	19.4 ± 1.1	75 ± 1.4 *	28.6 ± 1.9
PEEA-C3	60 ± 1.9	--	65 ± 1.7	--
RPEA-C5	--	--	75 ± 1.4 *	41.5 ± 1.1
Rocaglamide	100 ± 3.1 ***	0.03 ± 1.3		
Staurosporine			95 ± 1.15 ***	1.03 ± 1.2

Values (mean ± SD) are the average of three samples, each of which was analyzed individually in triplicate (*n* = 1 × 3). -- = No activity or not active. Results are highly significant ***, moderately significant **, and significant *.

## Data Availability

The data presented in this study are available on request from the corresponding author (the data are not publicly available due to the involvement of several institutions in this work and their varying policies).

## Supplementary Materials

The following supporting information can be downloaded at https://www.mdpi.com/article/10.3390/molecules30224339/s1; Figure S1. Plan for separating the fragments of EtOAc extract through Column Chromatography. Figure S2. Schematic Representation of isolation and purification of PEEA-C2 and PEEA-C. Figure S3. Structural elucidation of isolated Compounds RPEA-C5. Figure S4. MS spectra of PEEA-C2 compound (Molecular ion at (M+Na), (C_30_H_50_O; Mol Wt.426.73). Figure S5. 1D-NMR, ^13^C NMR spectra (400 MHz in CDCl_3_) of PEEA-C2 Compound. Figure S6. 1D-NMR, ^1^ H NMR spectra (400 MHz in CDCl_3_) of PEEA-C2 Compound. Figure S7. NMR Characterization of PEEA-C2. Figure S8. MS spectra of PEEA-C3 Compound (Molecular ion at (M+Na), (C_30_H_51_O; Mol Wt.426.72). Figure S9. 1D-NMR, ^1^H NMR spectra (400 MHz in CDCl_3_) of PEEA-C3 Compound. Figure S10. HSQC spectra obtained from 2D-NMR at 400MHz in CDCl3 for PEEA-C3 Compound. Figure S11. 1D-NMR, ^1^ H NMR spectra (400 MHz in CDCl_3_) of PEEA-C5 Compound. Figure S12. 1D-NMR, ^13^C NMR spectra (400 MHz in CDCl_3_) of PEEA-C5 Compound.
