# Supplementary material for: Bioactivity-Directed Isolation of Anticancer Constituents from Underexplored Folklore: Rhus punjabensis Stewart
Source: Molecules. 2025 Nov 8;30(22):4339. doi: 10.3390/molecules30224339 (PMC12655016; doi:10.3390/molecules30224339)
Supplement: Supplementary file 1 [file molecules-30-04339-s001.zip › molecules-3915302-supplementary.pdf]

Supplementary File

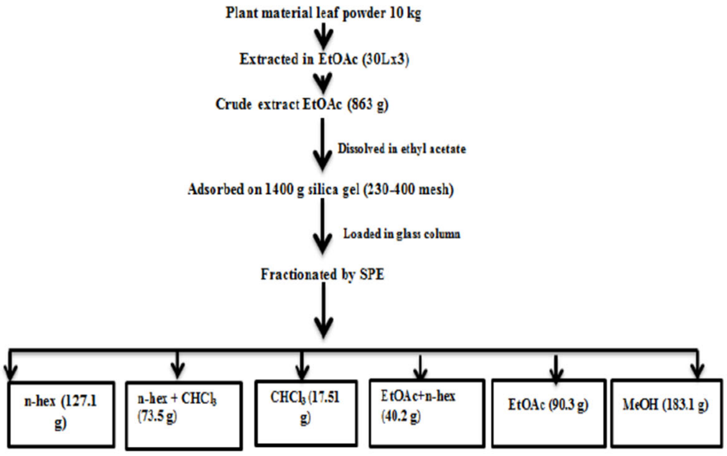

Figure S1. Plan for separating the fragments of EtOAc extract through Column Chromatography

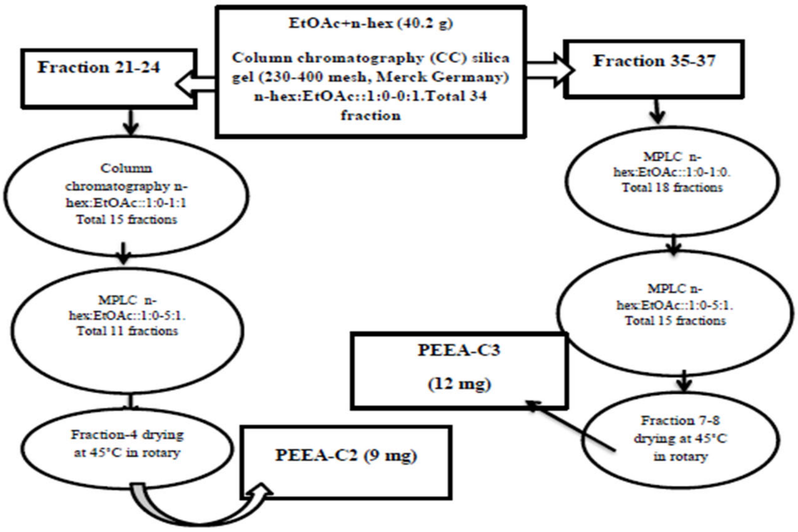

Figure S2. Schematic Representation of isolation and purification of PEEA-C2 and PEEA-C3

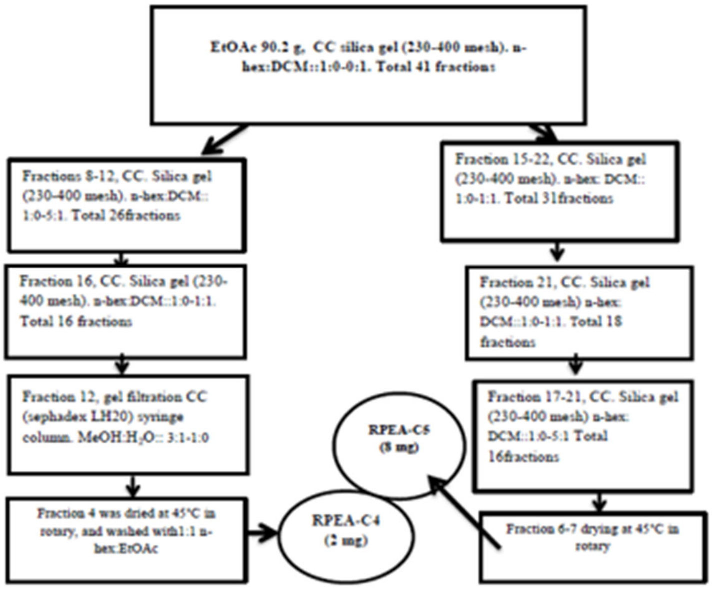

Figure S3. Structural elucidation of isolated Compounds RPEA-C5

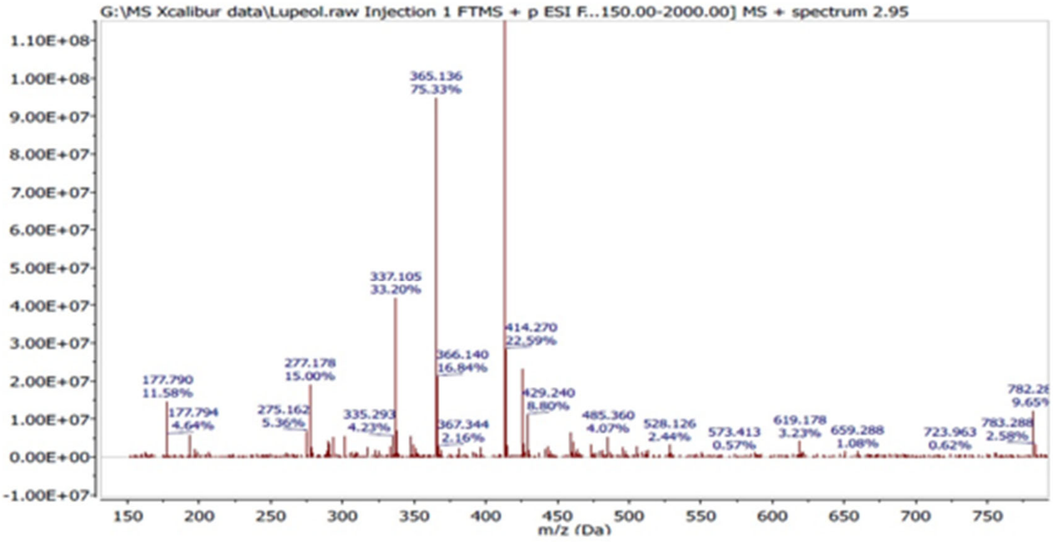

Figure S4. MS spectra of PEEA-C2 Compound (Molecular ion at (M+Na), ($C_{30}H_{50}O$; Mol Wt.426.73)

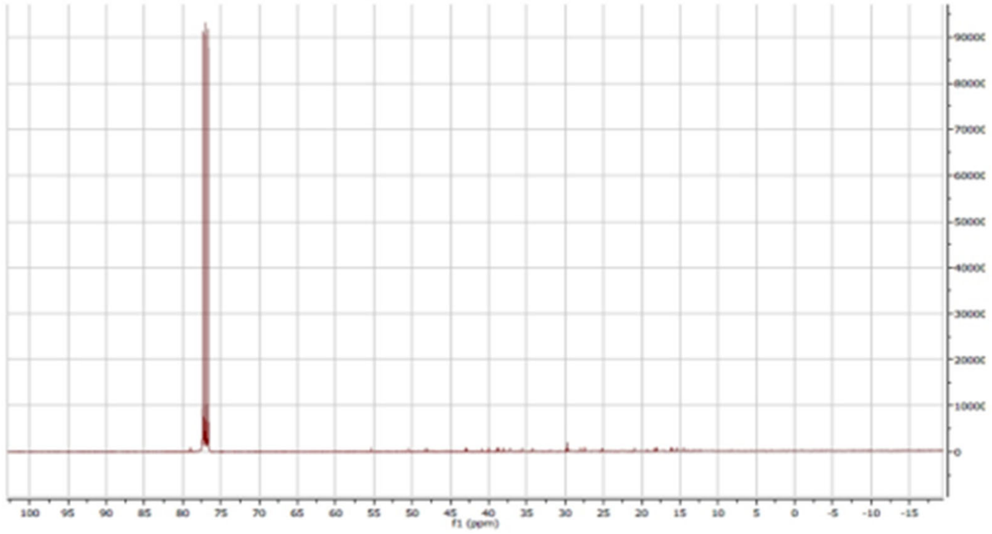

Figure S5. 1D-NMR, ^{13}C NMR spectra (400 MHz in $CDCl_3$) of PEEA-C2 Compound

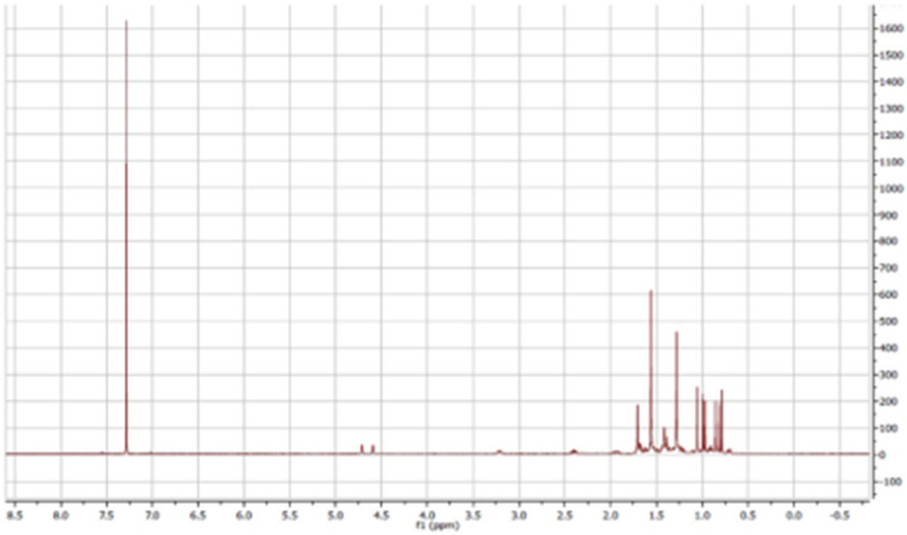

Figure S6. 1D-NMR, ^{1}H NMR spectra (400 MHz in $CDCl_3$) of PEEA-C2 Compound

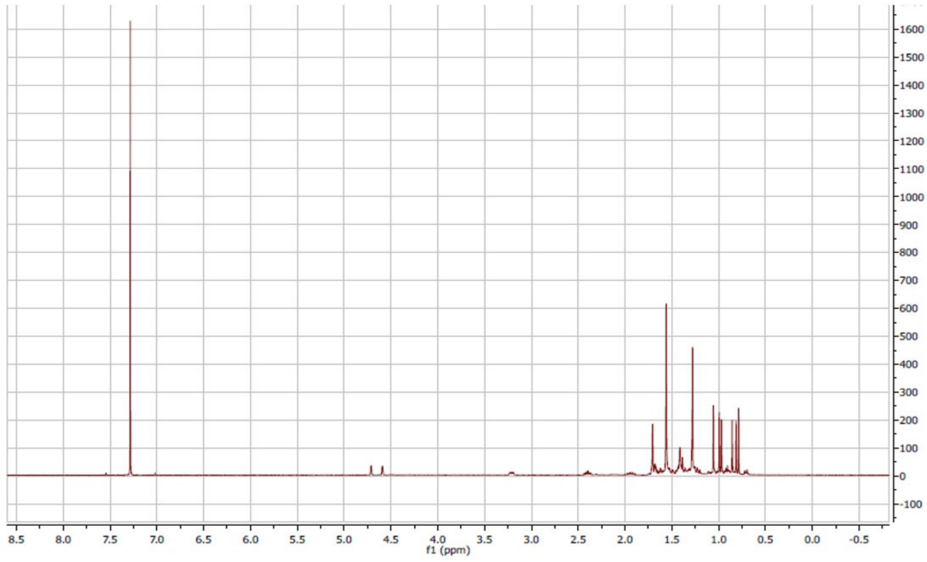

Figure 5: NMR characterization of PEEA-C2

Figure S7. NMR Characterization of PEEA-C2

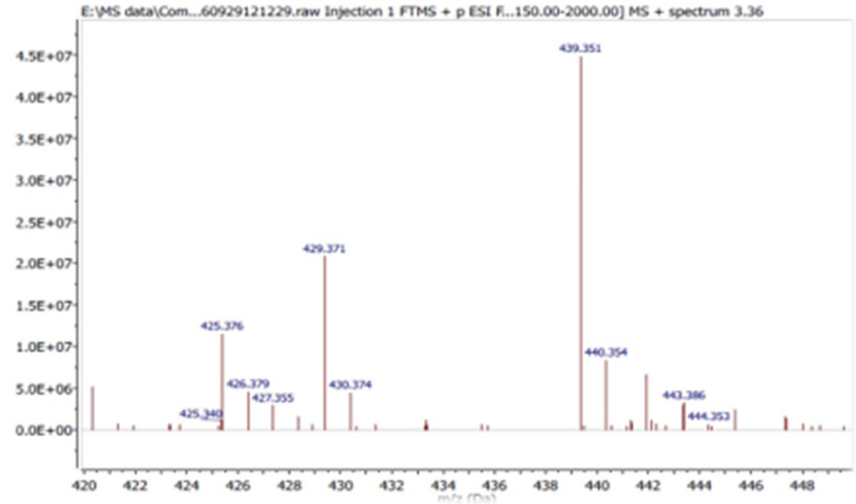

Figure S8. MS spectra of PEEA-C3 Compound (Molecular ion at (M+Na), ($C_{30}H_{51}O$; Mol Wt.426.72)

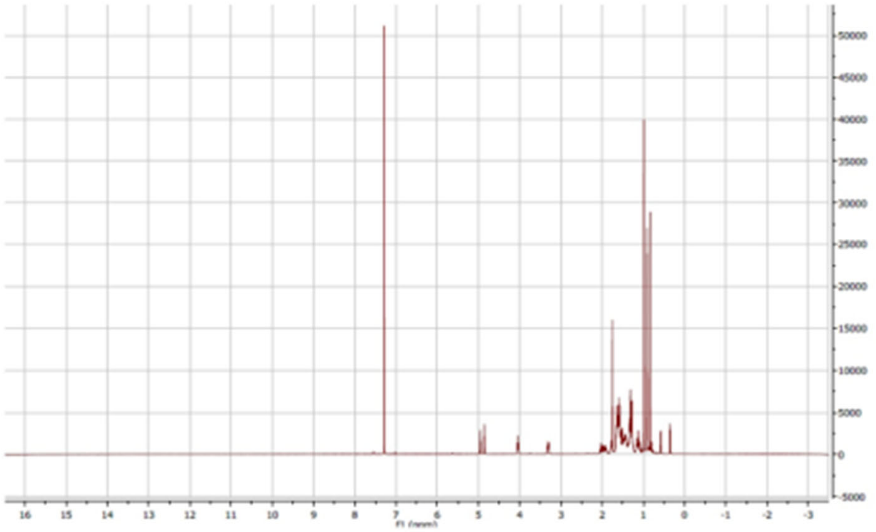

Figure S9. 1D-NMR, ^{1}H NMR spectra (400 MHz in $CDCl_3$) of PEEA-C3 Compound

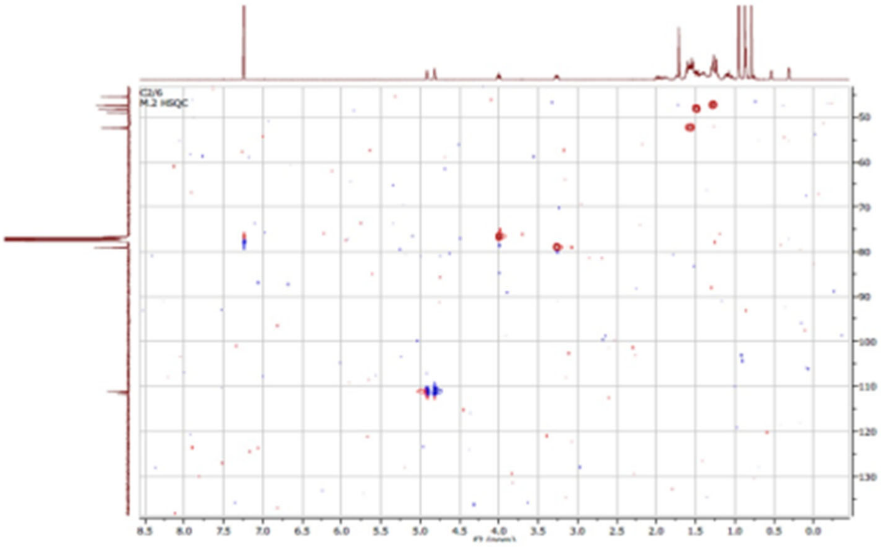

Figure S10. HSQC spectra obtained from 2D-NMR at 400MHz in CDCl3 for PEEA-C3 Compound

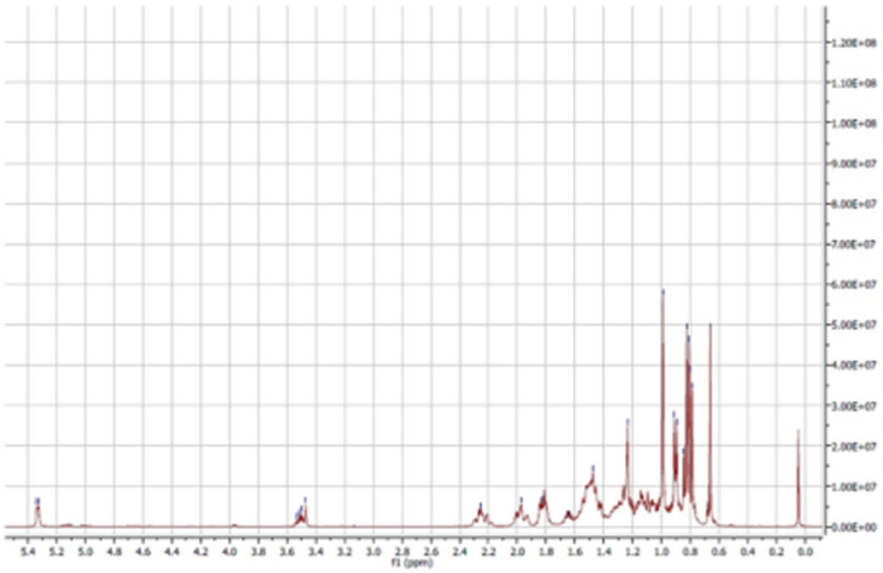

Figure S11. 1D-NMR, ^{1}H NMR spectra (400 MHz in $CDCl_3$) of PEEA-C5 Compound

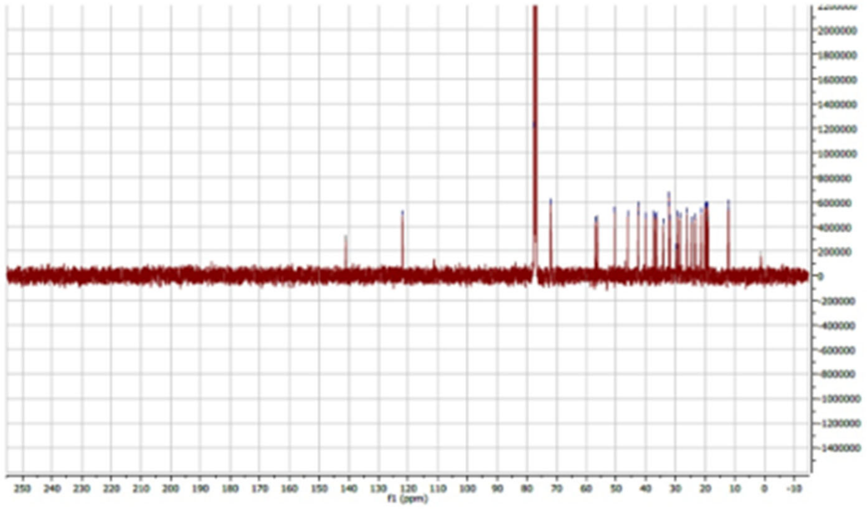

Figure S12. 1D-NMR, ^{13}C NMR spectra (400 MHz in $CDCl_3$) of PEEA-C5 Compound
